# Prevalence of hypertension and associated risks in hospitalized patients with COVID-19: a meta-analysis of meta-analyses with 1468 studies and 1,281,510 patients

**DOI:** 10.1186/s13643-022-02111-2

**Published:** 2022-11-17

**Authors:** Yousof Khairy, Deniz Naghibi, Ahmad Moosavi, Mehran Sardareh, Saber Azami-Aghdash

**Affiliations:** 1 Center for the Development of Interdisciplinary Research in Islamic Sciences and Health Sciences, Tabriz University of Medical Sciences, Tabriz, Iran; 2 Department of Public Health Sciences, School of Medicine and Dentistry, University of Rochester, Rochester, New York USA; 3 Department of Health and Community Medicine, Dezful University of Medical Sciences, Dezful, Iran; 4 Student Research Committee, Tabriz University of Medical Sciences, Tabriz, Iran; 5 Tabriz Health Services Management Research Center, Tabriz University of Medical Sciences, Tabriz, Iran

**Keywords:** Comorbid disease, COVID-19, Hypertension, Meta-analysis, Risk factor

## Abstract

**Background:**

Since the COVID-19 outbreak, preliminary research has shown that some risk-associated conditions increase death and severe complications of the disease, hypertension being one of them. Thus, numerous meta-analyses have been conducted to explore this issue. Therefore, this umbrella review aims to perform a meta-analysis of the meta-analyses to estimate the prevalence and associated risks of hypertension in patients with COVID-19.

**Methods:**

PubMed, Scopus, Web of Knowledge, Embase, and Cochrane databases were searched for the published meta-analyses up to January 1, 2022. Google Scholar, citation check, reference check, and Grey literature were also manually searched. A random-effect model approach was used for analysis.

**Results:**

The overall death rate was estimated at 12%. Hypertension was present in 25% of the patients as a comorbid disease. The overall RR for death, disease severity, and the possibility of ICU admission were estimated at 1.79 [1.68–1.89 with 95% CI], 1.74 [1.66–1.83 with 95% CI], and 1.91 [1.48–2.34 with 95% CI], respectively. The meta-regression results showed that being “male” significantly increases the risk of disease severity and ICU admission.

**Conclusions:**

The results indicated that hypertension is a common comorbid disease in hospitalized patients with COVID-19, which significantly increases mortality risk, the severity of the disease, and the probability of ICU admission.

**Systematic review registration:**

This study has been registered in PROSPERO (CRD42021231844).

**Supplementary Information:**

The online version contains supplementary material available at 10.1186/s13643-022-02111-2.

## Background

COVID-19 is an infectious disease caused by a novel coronavirus. It is a highly contagious disease from Wuhan, Hubei Province, China, and has spread to over 200 countries [1–4]. The most common symptoms are fever, dry cough, shortness of breath, weakness, and the loss of smell [5, 6]. It can range from a mild infection to acute respiratory distress syndrome (ARDS), similar to severe respiratory syndrome coronavirus (SARS-CoV) and Middle East respiratory syndrome coronavirus (MERS-CoV), which emerged in most countries around the world over the last 20 years [1, 2, 7]. COVID-19 rapidly became a worldwide emergency crisis [8, 9].

Based on the results of published studies and reports, a significant number of high-risk individuals and groups, whose mortality risk and severe complications are higher than others, have been identified since the onset of the COVID-19 pandemic [10–16]. Patients with hypertension belong to one of these high-risk groups [17–19]. Since there is an interaction between COVID-19 and Angiotensin-Converting Enzyme 2 (ACE2), it has been suggested that hypertension could play a role in pathogenesis COVID-19. It can either act as a direct clinical predictor of disease severity or aid in disease exacerbation at the end of a defined disease period through acute respiratory distress syndrome (ARDS), systemic inflammatory response syndrome (SIRS), or multiple organ failure (MOF) [20]. Hypertension is one of the most common conditions globally, leading to dangerous complications like acute heart attacks, heart diseases, and strokes. Therefore, healthcare systems face many treatment and caring challenges regarding these complications [21–24]. The number of patients with hypertension is growing, especially among the elderly population. It is estimated that by 2025, about 29% of the world’s population will have hypertension, and about 1.58 billion of the world’s adult population will suffer from the complications of this condition [25]. After about 2 years since the coronavirus outbreak, many studies have been conducted on the prevalence and effects of hypertension on patients with COVID-19.

Additionally, some researchers started conducting systematic reviews and meta-analyses using the findings of the studies [18, 19, 26, 27]. Although these studies have presented comprehensive and helpful information, it seems that study results differ from one another. On the other hand, due to the large number of these studies, decision-makers and managers face difficulties choosing which study to base their decisions on. Therefore, it appears that by summarizing and conducting a systematic meta-analysis of meta-analyses, not only will this study overcome these challenges, but because of the large sample size, it will also provide more reliable information for decision-makers, policy-makers, healthcare providers, and other readers. Thus, the present study is carried out to estimate the prevalence and severity of hypertension as a risk factor in patients with COVID-19.

## Methods

The present study is an umbrella review designed and conducted in 2021 to estimate the prevalence and severity of hypertension as a risk factor in patients with COVID-19. Preferred reporting items for systematic reviews and meta-analyses (PRISMA) [28] was used in this study. All procedures performed in this study are in accordance with the ethical standards of the institutional and national research committee. Institutional review board (IRB) approval has been obtained from the Research Ethics Committee of Tabriz University of Medical Sciences (ethics code IR.TBZMED.REC.1398.223). In addition, the protocol of this study has been registered in PROSPERO (registration code CRD42021231844).

### Search strategy

The search strategy in this study was developed and implemented by a highly knowledgeable librarian experienced in the related field (Additional file 1). PubMed, Scopus, Web of Knowledge, Embase, and Cochrane were searched using the relevant MeSH keywords up to January 1, 2022. Subsequently, some of the relevant journals as well as the search engine Google Scholar were manually searched to discover more articles. The articles were screened considering title/abstracts and full texts, respectively. Excluding the studies that met the exclusion criteria, researchers carried out a citation check (through Google Scholar citations), reference check (done manually from the reference list of the articles), and Grey literature search (through the European Association for Gray Literature Exploitation (EAGLE) and the Healthcare Management Information Consortium (HMIC) databases) to enhance the identification of the existing articles.

### Inclusion and exclusion criteria

#### Inclusion criteria

- All the studies and reports published in English worldwide that had analyzed the prevalence and severity of hypertension as a risk factor in patients with COVID-19 using meta-analysis

#### Exclusion criteria

- The studies without meta-analysis, such as narrative reviews and extensive reviews
- The studies which had not to mention the prevalence or effects (disease severity, death, ICU admission) of hypertension
- The studies which had mentioned the effects of interventions and medications
- The studies and reports that lacked a full text or the ones whose full texts could not be accessed
- The articles with poor reporting quality (a score lower than 5 out of 11 in reporting quality assessment)
- The articles whose target group was merely the deceased patients
- The meta-analyses in which the included studies were not sufficient or the ones with an inappropriate reporting method

### Assessment of articles’ reporting quality

The reporting quality of all articles in the full-text screening was assessed by two reviewers independently using the tool for assessment of multiple systematic reviews (AMSTAR) [29]. The responses associated with each item are indicated as “Yes”, “No”, “Cannot be answered”, or “non-applicable” in this tool. The answer of “Yes” was given a score of 1, and the answers of “No”, “Cannot be answered”, or “non-applicable” were given a score of 0. According to this tool, the articles with a score of 1 to 4, 5 to 8, and 9 to 11 are rated as “low quality”, “medium quality”, and “high quality”, respectively. The two reviewers’ agreement gave the final assessment score for each article. Disagreements between the two reviewers were referred to a third assessor.

### Data extraction

In order to extract data, a data extraction form was designed manually in Word 2013 Office software. The information form included the following information:

Author, year, first author’s country of affiliation, number of included articles in meta-analysis, latest search date (month), number of the total population reviewed in meta-analysis, target group (mean of age, male %, mortality %), pooled prevalence of hypertension % (95% CI), and risk ratio (RR) for death (*I*^2^%), severity (*I*^2^%), ICU admission (*I*^2^%).

First, the data of five randomly selected articles were extracted to pilot the form with the results of these studies being re-used in the main study; the shortcomings and defects of the initial form were removed. In some cases where the required information was not reported in the articles, the research team calculated it based on the characteristics of the articles included in the meta-analysis. In some other cases, an email was sent to the corresponding author to require the information. In some studies, the number of primary articles included in the systematic reviews differed from those in the quantitative analyses (meta-analyses). Accordingly, the researchers considered the number of articles included in the meta-analysis. The information reported in the articles was the basis for extracting RR data for disease severity and ICU admission. The research team did not make any decisions in this regard. For example, some studies reported ICU admission as a disease severity criterion, while other studies reported it separately.

### Data analysis methods

In order to estimate the prevalence of hypertension, mortality rate, and the impact of hypertension on patients with COVID-19, statistical meta-analysis methods and a random model were used. Stata (StataCorp, version 16) was used to perform meta-analysis [30]. Since in some studies RR and in the others odds ratio (OR) was reported, it was decided that OR would be converted to RR in the present research. To do this, the formula presented in Zhang et al.’s study [31] was used. In order to report the results, forest plot graphs were used, in which the size of each square indicated the sample volume, and the lines drawn on each side of the square showed a 95% confidence interval for each study. The indicator *I*^2^ was used to assess the heterogeneity of studies’ results. In this study, *I*^2^ lower than 50%, *I*^2^ between 50 and 74%, and *I*^2^ higher than 75% were considered low heterogeneity, medium heterogeneity, and high heterogeneity, respectively [32]. Meta-regression was carried out based on the mean variables of age (year), male percentage, and the latest reference searching date (month). The date was considered due to the galloping speed of publishing primary articles and subsequent systematic reviews as well as the spread of COVID-19 spread. Also, sub-group analyses were carried out based on the latest date of search (month). Funnel plot graph and Egger’s regression test were used at a significance level of 0.1 [33] to assess publication bias. The Trim and Fill test was used through a linear estimator whenever publication bias was potential.

For assessing the percentage of the overlap of primary studies, corrected covered area (CCA), and covered area (CA) introduced by Pieper et al. [34] was applied. Overlap was defined as primary studies that were included in more than one meta-analysis.

$$\textrm{Covered}\ \textrm{Area}\ \left(\textrm{CA}\right)=\frac{N}{rc}$$

$$\textrm{Corrected}\ \textrm{Covered}\ \textrm{Area}\ \left(\textrm{CCA}\right)=\frac{N-r}{rc-r}$$

*N*: The sum of primary published studies and repeated studies are counted to calculate *N*

*r*: Number of rows or index publication

*c*: Number of columns or reviews

## Results

**Fig. 1:**
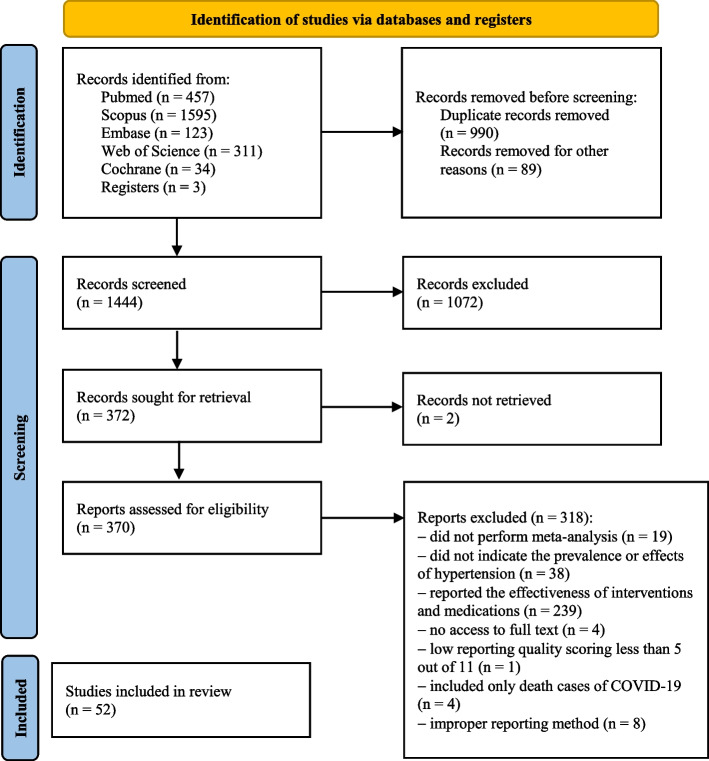
Searching and screening process (PRISMA Flowchart)

Out of 2523 records found from databases and other sources, 1079 studies were excluded due to duplication and meeting the exclusion criteria. A total of 1072 records were excluded when titles and abstracts were screened. In addition, 328 records were excluded after full-text screening. Finally, 52 meta-analyses were included [26, 35–85] (see Fig. 1 for PRISMA flowchart). Also, the PRISMA checklist is provided in the Additional file 2. The characteristics and results of the included meta-analyses are demonstrated in Additional file 3.

Twenty studies were initially based in China, and six were from the US. Fifty-two meta-analyses were reviewed, including 1468 studies and 1,281,510 patients with COVID-19. The latest date (month) of reference searching varied from the 2nd to the 11th month of 2020, in which most cases belonged to the 3rd and 4th months. The mean age of the patients studied in the articles was 53. In addition, about 60% of the study participants were males.

### Mortality rate in patients

**Fig. 2:**
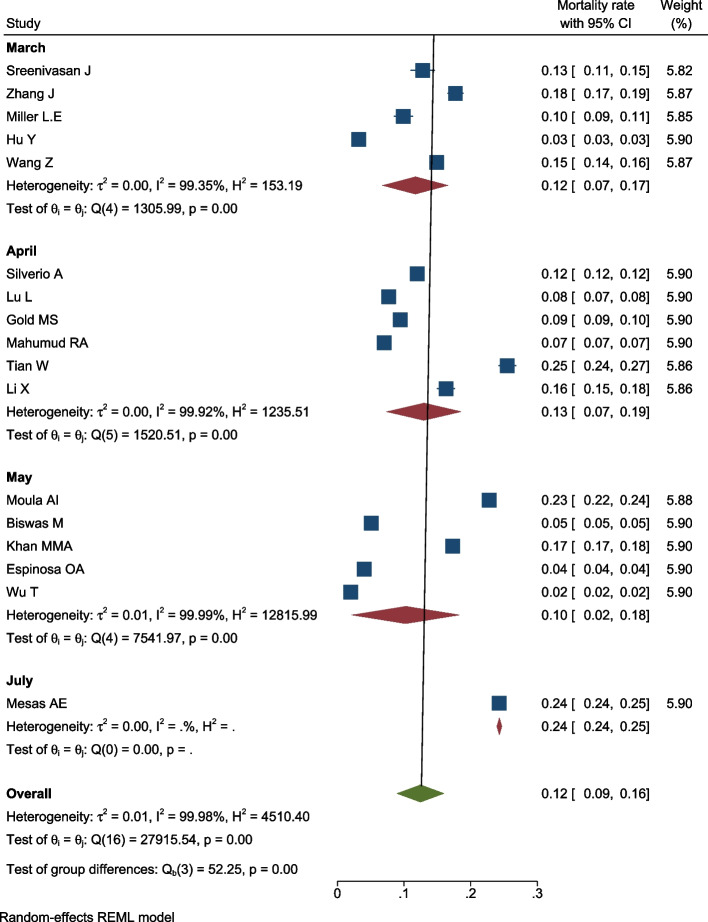
Mortality rate in COVID-19 patients based on the information from 17 meta-analyses with 741399 patients

In 17 studies with an overall sample size of 741,399 patients, the mortality rate in patients with COVID-19 was reviewed. Meta-analysis showed that the overall mortality rate was 12% [9–16% with 95% CI] (Fig. 2). Heterogeneity assessment results showed high heterogeneity in the results of the studies (for more information, refer to Table 1).

**Table 1:** Mortality rate, hypertension prevalence, RR for death, disease severity, and ICU admission among COVID-19 patients

Variable	Last date of search (month)	Number of studies	Summary estimates (prevalence/RR) [95% CI]	Heterogeneity (***I***^**2**^ %)	Publication bias-Egger test (***P*** value)
Mortality rate	March	5	12% [7–17%]	99.3	N/A
	April	6	13% [7–19%]	99.9	N/A
	May	5	10% [2–18%]	99.9	N/A
	July	1	24% [24–25%]	0.00	N/A
	Overall	17	12% [9–16%]	99.9	N/A
Hypertension prevalence	February	3	20% [17–22%]	86	N/A
	March	7	20% [15–25%]	99.3	N/A
	April	13	25% [19–32%]	99.9	N/A
	May	7	24% [17–31%]	99.9	N/A
	June	2	29% [23–35%]	99.6	N/A
	July	4	36% [26–47%]	99.8	N/A
	Overall	36	25% [22–28%]	99.9	N/A
RR for death	March	2	2.22 [1.47–2.98]	0.00	0.001
	April	8	1.84 [1.62–2.05]	77.8	
	May	6	1.88 [1.74–2.02]	0.00	
	June	1	1.83 [1.63–2.03]	–	
	July	2	1.78 [1.45–2.11]	0.00	
	September	1	1.74 [1.41–2.08]	–	
	November	1	1.49 [1.35–1.63]	-	
	Overall	21	1.79 [1.68–1.89]	61.5	
RR for disease severity	February	3	1.87 [1.63–2.11]	0.00	0.007
	March	7	1.72 [1.45–1.99]	50.2	
	April	10	1.77 [1.62–1.93]	31.5	
	May	4	1.89 [1.63–2.15]	0.00	
	July	2	1.62 [1.25–2.00]	0.00	
	Overall	30	1.74 [1.66–1.83]	27.7	
RR for ICU admission^a^	Overall	5	1.91 [1.48–2.34]	74.9	0.462

*CI* Confidence Interval

^a^Due to the small number of studies in this section, the subgroup analysis was not performed

### Prevalence of hypertension (as a comorbid disease)

**Fig. 3:**
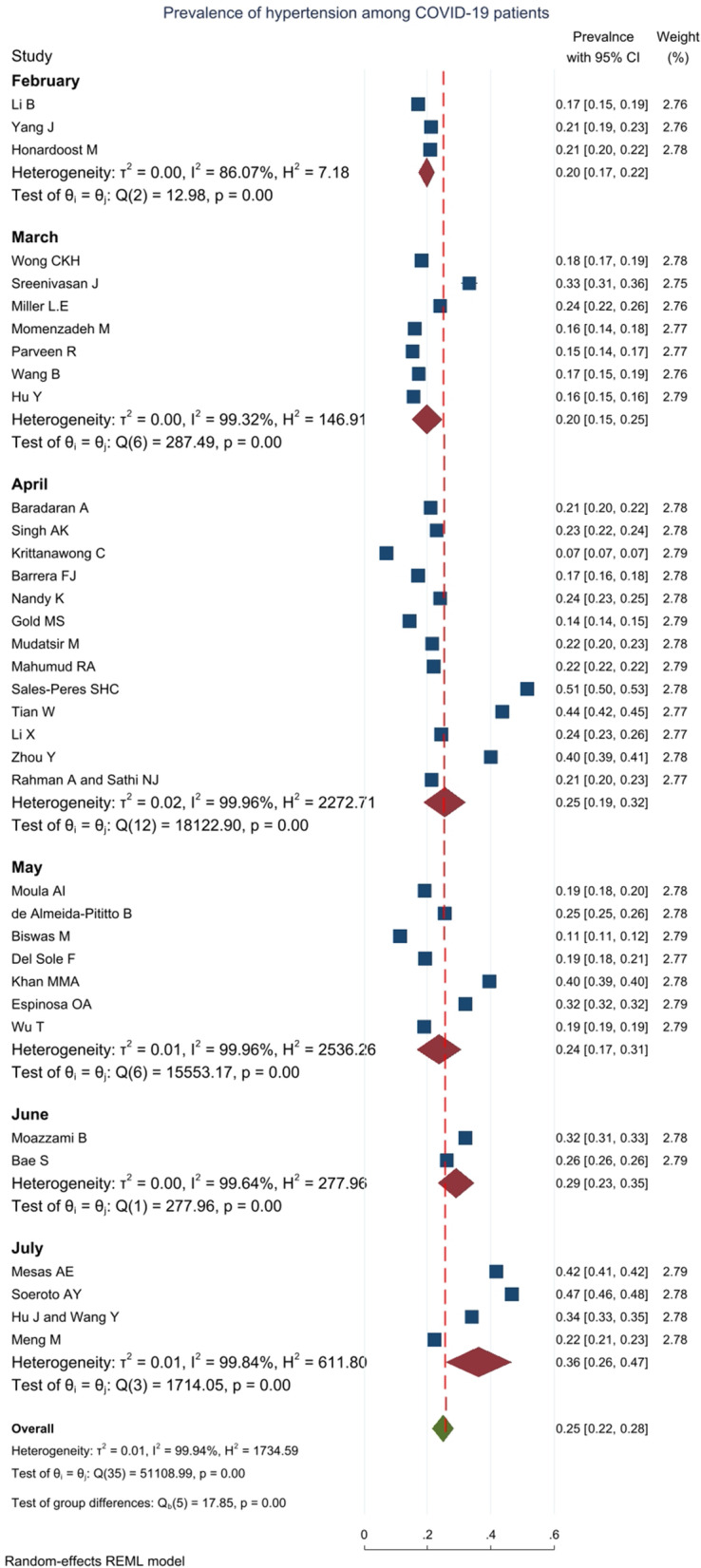
Prevalence of hypertension among COVID-19 patients based on 33 meta-analyses with 904104 patients

In 36 studies with an overall sample size of 960,963 patients, the prevalence of hypertension as a comorbid disease was reported, and meta-analysis results showed that the overall prevalence percentage was 25% [22–26% with 95% CI] (Fig. 3). Heterogeneity assessment results showed high heterogeneity in the results of the studies (for more information refer to Table 1).

### RR for death

**Fig. 4:**
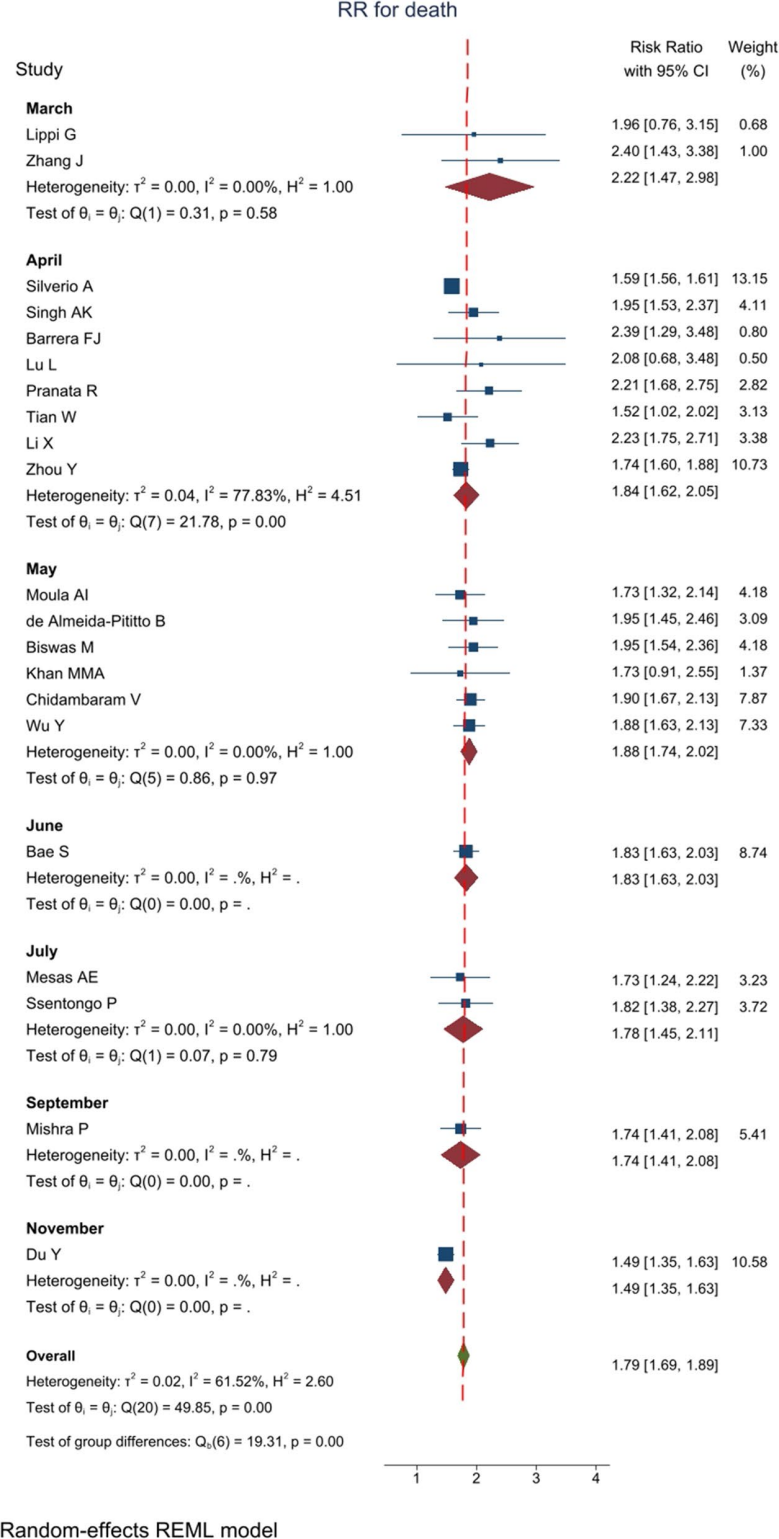
The RR impact of hypertension on patients’ death based on 17 meta-analyses with 368284 patients

**Fig. 5:**
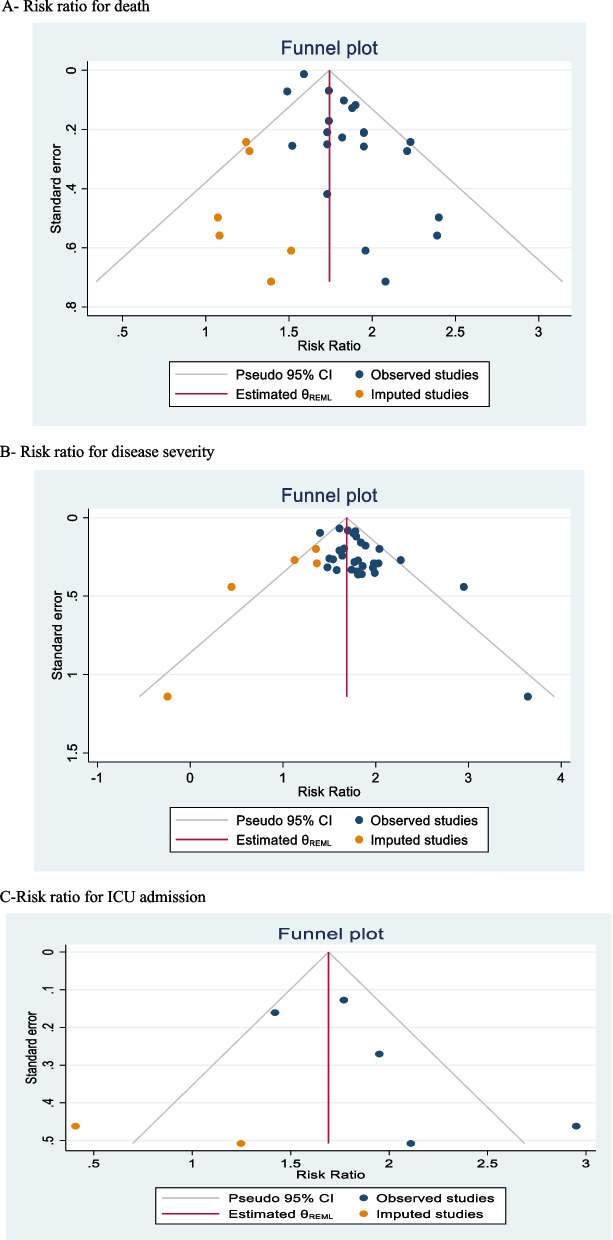
Funnel plot graphs. **A** Risk ratio for death. **B** Risk ratio for disease severity. **C** Risk ratio for ICU admission

In 21 studies with an overall sample size of 548,776 patients, RR for hypertension was reported in the death of patients with COVID-19. Meta-analysis results showed that the overall RR was 1.79 [1.69–1.89 with 95% CI] (Fig. 4). Heterogeneity assessment results showed a medium heterogeneity in the results of the studies. The results of assessing the potential for publication bias showed a high possibility of publication bias in the studies’ findings (for more information, refer to Table 1) (Fig. 5A). Furthermore, the Trim and Fill test results showed that six studies are possibly missing and that with the imputation of these studies and their effect, RR decreases to 1.74 [1.64–1.84 with 95% CI]. The mean *I*^2^ of meta-analyses in this section was 58.4%.

### RR for disease severity

**Fig. 6:**
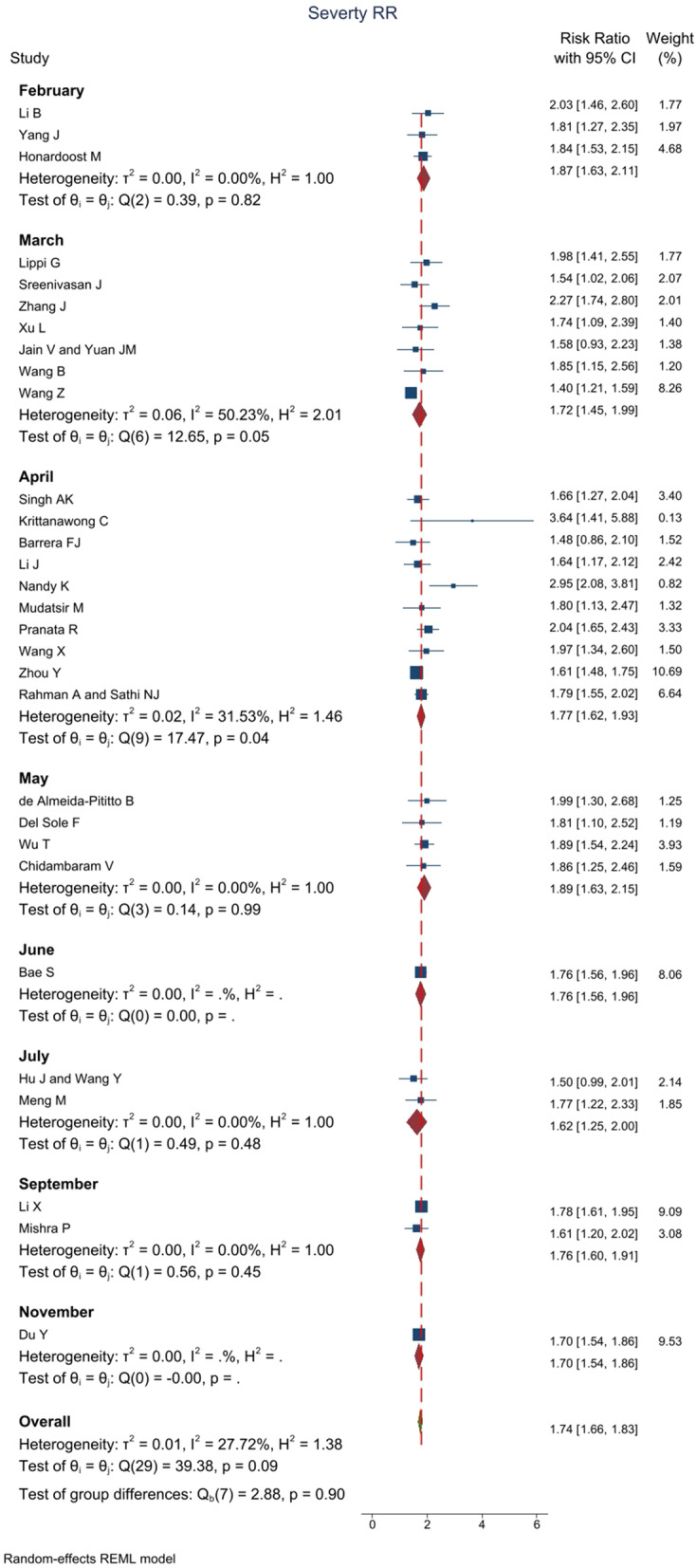
The RR impact of hypertension on disease severity among COVID-19 patients based on 24 meta-analyses with 383794 patients

In 30 studies with an overall sample size of 568,503 patients, RR for hypertension in the disease severity of patients with COVID-19 was reported. Meta-analysis results showed that the overall RR was 1.74 [1.66–1.83 with 95% CI] (Fig. 6). Heterogeneity assessment results showed a low heterogeneity in the results of the studies. The results of assessing the potential for publication bias showed a high possibility of publication bias in the studies’ findings (for more information, refer to Table 1) (Fig. 5B). Furthermore, the Trim and Fill test results showed that six studies are possibly missing, and with the imputation of these studies and their effect, RR decreases to 1.68 [1.63–.74 with 95% CI]. The mean *I*^2^ of meta-analyses in this section was 49.6%.

### RR for ICU admission

**Fig. 7:**
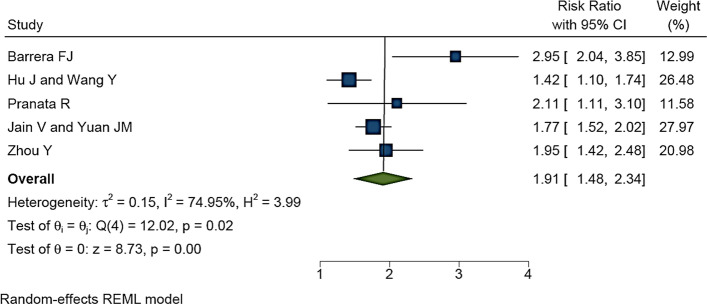
The RR impact of hypertension on hospitalization of COVID-19 patients in ICU based on five meta-analyses with 43079 patients

In five studies with an overall sample size of 43079 patients, RR for hypertension in the patients with COVID-19 hospitalized in ICU was reported. Meta-analysis results showed that the overall RR was 1.91 [1.48–2.34 with 95% CI] (Fig. 7). Heterogeneity assessment results showed a medium heterogeneity in the results of the studies. The results of assessing the potential for publication bias showed a very low possibility of publication bias in the studies results (for more information, refer to Table 1) (Fig. 5C). The mean of I^2^ of meta-analyses in this section was 36.1%.

### Meta-regression results

Meta-regression results with a random model, based on the patients’ mean age (year), male percentage, and the latest reference searching date (month), showed that among these variables, only the male percentage significantly estimates RR for disease severity and ICU admission. Furthermore, a 1% increase in the ratio of male proportion causes the RR value for disease severity to increase by almost 0.03 in contrast to RR for ICU admission, which decreases by almost 0.13. None of these variables were significant predictors of patient deaths (Table 2).

**Table 2:** Meta-regression models adjusted for prediction of death, disease severity, and ICU admission of COVID-19 patients

Outcomes	Variable	Number of studies	Regression coefficient [95% CL]	***P*** value	***I***^**2**^%
Death	Mean of age	9	− 0.007 [− 0.059, 0.044]	0.72	11.5
	Male %	12	− 0.065 [− 0.158, 0.026]	0.13	0.00
	Last date of search (month)	21	− 0.014 [− 0.146, 0.117]	0.82	53.9
Disease severity	Mean of age	14	0.016 [− 0.039, 0.072]	0.52	49.6
	Male %	19	0.030 [0.009, 0.051]	**0.009**	0.00
	Last date of search (month)	30	− 0.009 [− 0.113, 0.094]	0.86	39.2
ICU admission	Mean of age	3	− 0.082 [− 0.785, 0.621]	0.81	0.00
	Male %	4	− 0.136 [− 0.218, 0.053]	**0.001**	0.00
	Last date of search (month)	5	− 0.148 [− 0.448, 0.151]	0.33	66.2

### Results of articles reporting quality assessment

One study was excluded for low reporting quality (lower than 5 out of 11). The reporting quality was medium (5 to 8) in 19 and high (9 to 11) in 31 studies. The median reporting quality score was 9 (with a mean of 8.6 out of 11) (Additional file 4).

### Results of overlap assessment

The overlap calculation results indicate a slight percent of overlap (CA 4.24% and CCA 2.44%) (Additional file 5).

## Discussion

The results of 52 meta-analyses of 1468 articles with 1281510 COVID-19 patients were reviewed. The overall mortality rate in patients with COVID-19 was estimated at 12%. It was estimated that hypertension was a comorbid disease in 25% of the patients. The overall RR for hypertension in the death of patients with COVID-19, disease severity, and probability of ICU admission was estimated at 1.79 [1.68–1.89 with 95% CI], 1.74 [1.66–1.83 with 95% CI], and 1.91 [1.48–2.34 with 95% CI], respectively. In general, reporting quality of the articles was assessed as appropriate.

After studying the latest date of article search, it was revealed that the latest search in meta-analyses was conducted on the 11th month of 2020. In other words, the results of the articles published after the 11th month did not undergo systematic review and meta-analysis (or they have not been published yet). Considering high speed and large amount of the articles published on this subject, fast changes in prevalence, complications, and other aspects of COVID-19, and consequent changes in the study results, it seems that the results of the new studies have to be gathered and reported using meta-analysis, just like the first months after coronavirus outbreak. During this period, plenty of articles and evidence were rapidly published, and different researchers gathered, assessed, combined and reported their results in the form of meta-analyses. However, this should not cause researchers and editors-in-chief to neglect the quality of meta-analyses, for the researchers may not have enough time to conduct high quality studies. Given the pressures to rapidly publish articles on COVID-19, editors-in-chief and reviewers might neglect the quality.

According to the current study results, the overall mortality rate in patients with COVID-19 was 12%. According to the latest update of WHO statistics (4:14 pm CET, December 29 2021), 281,808,270 cases have been confirmed as testing positive for COVID-19, 5,411,759 of whom have died, so the mortality percentage is about 1.92 [86]. However, the studies that calculated the mortality rate of hospitalized patients have reported a figure close to the present study [87–90]. Nevertheless, some significant changes are seen in the mortality rate in different studies, the reason for which can be the type of treatment and medical services, demographic features of patients, various risk factors in different countries, and the advances obtained over time on the treatment. The critical point, in this regard, is to remove the mistakes and ambiguities of calculations, timely and appropriate reporting, and transparency of statistics, which the countries must consider to have proper decision-making and planning.

The study results revealed that a high percentage of patients with COVID-19 (25%) had hypertension as a comorbid disease. Likewise, the results of most studies undergoing meta-analysis showed that hypertension is the most common comorbid disease in patients with COVID-19. One of the probable reasons could be the prevalence of hypertension in society, especially among adults. Similarly, according to global evidence and reports, the prevalence of hypertension among people worldwide is high and closer to the results of this study (among the hospitalized patients with COVID-19) [91–95]. Considering the high prevalence of hypertension in society and its high risk in the mortality and unpleasant complications of COVID-19, serious and immediate interventions must be carried out to control hypertension in patients beside preventing hypertensive patients from contracting COVID-19 since the simultaneity of these two issues, suffering from hypertension and COVID-19, has unpleasant clinical complications, and it can have negative social and economic effects on patients, healthcare systems, and governments [96].

The results showed that, in general, hypertension increases these patients’ risk of death, risk of developing another severe disease, and the risk of ICU admission by 79%, 74%, and 91%, respectively. According to the previous studies, various reasons are mentioned for the effects of hypertension on patients with COVID-19, including higher neutrophil-lymphocyte ratio [97, 98], and higher D-dimer levels [99], and higher C-reactive protein [100]. Furthermore, there is ample evidence that patients treated with ACE (angiotensin-converting enzyme) inhibitors are at a higher risk of death and severe complications than others [20, 101–103]. Although the available evidence cannot claim with certainty regarding the impact and the reasons for the adverse effects of hypertension on patients with COVID-19, it appears that a strong and large body of evidence confirms the high risk of mortality and severity of COVID-19 in hypertensive patients, as the results of this study with a large sample size indicated. Therefore, it is essential to pay special attention to this group of people by quarantining, social distancing, regularly controlling blood pressure, using highly effective masks, prioritizing receiving confirmed vaccines, and other controlling actions.

Meta-regression results showed that being male significantly increases disease severity and ICU admission. As a result, it is considered a risk factor, while it has a negative and insignificant effect on the death caused by COVID-19. Many studies and reports indicate a high risk of unpleasant complications and a high mortality rate among males [104–107]. Unlike most previously published studies [108–110], the present study could not show age as a risk factor determining death and disease severity. One of the main reasons the findings of this study are not in line with the findings of other studies could be the participants’ low mean age. In this study, the participants’ mean age was 53 years. However, in most published articles and reports, ages over 65 have been introduced as the leading risk factor for death [111–114]. On the other hand, considering the lack of information in some articles and the means reported in meta-analyses being close, it was impossible to group the means of age.

According to the results of text reviews and to the best of our knowledge, the present study is innovatively combining the results of the published meta-analyses on the prevalence and risk of hypertension in patients with COVID-19 (including the data from 1468 articles with 1,281,510 patients) for the first time. It provides transparent and comprehensive information for the decision-makers, healthcare providers, and other readers. Still, this study had some major limitations. The readers must pay attention to them while reading, interpreting, using the study results, and making cautious conclusions. One of the most important limitations concerns searching in English both in this study and in most other reviewed studies. The results could differ once published in other languages (especially Chinese) in the analysis. Another limitation of this study was that although the percentage of overlap of primary studies was negligible, removing repeated studies in the published meta-analyses was not possible because the researchers intended to use a combination of meta-analyses results. Furthermore, heterogeneity assessment results showed potential for publication bias, though the Trim and Fill test results indicated that its effect would not be significant if such bias occurs. However, it is recommended that the results be interpreted cautiously.

## Conclusion

The meta-analysis of meta-analyses consisting of 1468 articles with 1281510 patients with COVID-19 indicated that hypertension is a prevalent disease among hospitalized patients with COVID-19, which significantly increases the risk of death, and disease severity, and ICU admission. Therefore, it is essential to pay attention to this group of people by quarantining, social distancing, regularly controlling blood pressure, using highly effective masks, prioritizing receiving confirmed vaccines, and other controlling actions. However, the readers must be cautious in interpreting and using the study results.

## Supplementary Information

**Additional file 1.** Search strategy.

**Additional file 2.** PRISMA checklist.

**Additional file 3.** Extraction form for the data of the included studies.

**Additional file 4.** AMSTAR (Assessment of Multiple Systematic Reviews) checklist.

**Additional file 5.** Overlap calculation.

## Data Availability

The datasets used and analyzed during the current study are available from the corresponding author on reasonable request (s.azami.a90@gmail.com).

## Abbreviations

ACE2: Angiotensin-converting enzyme 2
ARDS: Acute respiratory distress syndrome
CI: Confidence interval
COVID-19: Coronavirus disease 2019
ICU: Intensive care unit
MOF: Multiple organ failure
OR: Odds ratio
RR: Risk ratio
SIRS: Systemic inflammatory response syndrome
WHO: World Health Organization
