# Supplementary material for: Prevalence of hypertension and associated risks in hospitalized patients with COVID-19: a meta-analysis of meta-analyses with 1468 studies and 1,281,510 patients
Source: Syst Rev. 2022 Nov 17;11:242. doi: 10.1186/s13643-022-02111-2 (PMC9672558; doi:10.1186/s13643-022-02111-2)
Supplement: Supplementary file 1 — Additional file 1. Search strategy. [file 13643_2022_2111_MOESM1_ESM.doc]

| **Complete search strategy for the databases** | |
| --- | --- |
| **PubMed** | |
| **457** | (("hypertension "[Title/Abstract] OR " blood pressure " AND ("2019 novel coronavirus"[Title/Abstract] OR "COVID19"OR "COVID-19"[Title/Abstract] OR "COVID 2019"OR "2019-novel CoV"[Title/Abstract] OR "SARS-cov-2"[Title/Abstract] OR "SARS-CoV2"OR "SARSCoV2"OR "SARSCoV-2"[Title/Abstract] OR "severe acute respiratory syndrome coronavirus 2"[Title/Abstract] OR "2019-ncov"OR "coronavirus disease 2019"OR "coronavirus disease-19"[Title/Abstract] OR "2019ncov"[Title/Abstract] OR "SARS coronavirus 2"[Title/Abstract] OR "severe acute respiratory syndrome coronavirus 2"[Title/Abstract] OR "COVID-19"[Title/Abstract])) AND ("review"[Title/Abstract] OR "systematic review"[Title/Abstract] OR "meta analysis"[Title/Abstract]) |
| **Scopus** | |
| **1,595** | ( "2019 novel coronavirus"  OR  "COVID19"  OR  "COVID-19"  OR  "COVID 2019"  OR  "2019-novel CoV"  OR  "SARS-cov-2"  OR  "SARS-CoV2"  OR  "SARSCoV2"  OR  "SARSCoV-2"  OR  "severe acute respiratory syndrome coronavirus 2"  OR  "2019-ncov"  OR  "coronavirus disease 2019"  OR  "coronavirus disease-19"  OR  "2019ncov"  OR  "SARS coronavirus 2"  OR  "severe acute respiratory syndrome coronavirus 2"  OR  "COVID-19" )  AND  ( "review"  OR  "systematic review"  OR  "meta analysis" )  AND  ( hypertension  OR  blood  AND pressure )  AND  ( LIMIT-TO ( LANGUAGE ,  "English" ) )  AND  ( LIMIT-TO ( DOCTYPE ,  "ar" )  OR  LIMIT-TO ( DOCTYPE ,  "re" ) )  AND  ( LIMIT-TO ( SUBJAREA ,  "MEDI" )  OR  LIMIT-TO ( SUBJAREA ,  "NURS" )  OR  LIMIT-TO ( SUBJAREA ,  "MULT" )  OR  LIMIT-TO ( SUBJAREA ,  "HEAL" ) ) |
| **Embase** | |
| **123** | hypertension "[Title/Abstract] OR " blood pressure " AND ("2019 novel coronavirus"[Title/Abstract] OR "COVID19"OR "COVID-19"[Title/Abstract] OR "COVID 2019"OR "2019-novel CoV"[Title/Abstract] OR "SARS-cov-2"[Title/Abstract] OR "SARS-CoV2"OR "SARSCoV2"OR "SARSCoV-2"[Title/Abstract] OR "severe acute respiratory syndrome coronavirus 2"[Title/Abstract] OR "2019-ncov"OR "coronavirus disease 2019"OR "coronavirus disease-19"[Title/Abstract] OR "2019ncov"[Title/Abstract] OR "SARS coronavirus 2"[Title/Abstract] OR "severe acute respiratory syndrome coronavirus 2"[Title/Abstract] OR "COVID-19"[Title/Abstract])) AND ("review"[Title/Abstract] OR "systematic review"[Title/Abstract] OR "meta analysis"[Title/Abstract]) |
| **Web of Science** | |
| **311** | hypertension "[Title/Abstract] OR "blood pressure" AND ("2019 novel coronavirus"[Title/Abstract] OR "COVID19"OR "COVID-19"[Title/Abstract] OR "COVID 2019"OR "2019-novel CoV"[Title/Abstract] OR "SARS-cov-2"[Title/Abstract] OR "SARS-CoV2"OR "SARSCoV2"OR "SARSCoV-2"[Title/Abstract] OR "severe acute respiratory syndrome coronavirus 2"[Title/Abstract] OR "2019-ncov"OR "coronavirus disease 2019"OR "coronavirus disease-19"[Title/Abstract] OR "2019ncov"[Title/Abstract] OR "SARS coronavirus 2"[Title/Abstract] OR "severe acute respiratory syndrome coronavirus 2"[Title/Abstract] OR "COVID-19"[Title/Abstract])) AND ("review"[Title/Abstract] OR "systematic review"[Title/Abstract] OR "meta analysis"[Title/Abstract]) |
| **Cochrane** | |
| **34** | hypertension"[Title/Abstract] OR "blood pressure" AND ("2019 novel coronavirus"[Title/Abstract] OR "COVID19"OR "COVID-19"[Title/Abstract] OR "COVID 2019"OR "2019-novel CoV"[Title/Abstract] OR "SARS-cov-2"[Title/Abstract] OR "SARS-CoV2"OR "SARSCoV2"OR "SARSCoV-2"[Title/Abstract] OR "severe acute respiratory syndrome coronavirus 2"[Title/Abstract] OR "2019-ncov"OR "coronavirus disease 2019"OR "coronavirus disease-19"[Title/Abstract] OR "2019ncov"[Title/Abstract] OR "SARS coronavirus 2"[Title/Abstract] OR "severe acute respiratory syndrome coronavirus 2"[Title/Abstract] OR "COVID-19"[Title/Abstract])) AND ("review"[Title/Abstract] OR "systematic review"[Title/Abstract] OR "meta analysis"[Title/Abstract] |
