# Supplementary material for: Prevalence of hypertension and associated risks in hospitalized patients with COVID-19: a meta-analysis of meta-analyses with 1468 studies and 1,281,510 patients
Source: Syst Rev. 2022 Nov 17;11:242. doi: 10.1186/s13643-022-02111-2 (PMC9672558; doi:10.1186/s13643-022-02111-2)
Supplement: Supplementary file 3 — Additional file 3. Extraction form for the data of the included studies. [file 13643_2022_2111_MOESM3_ESM.docx]

| **Author: year, Country** | **Number of included articles in MA** | **Last date of search** | **Number of the total population reviewed in MA (number of Hypertension)** | **Target group** | | | **pooled Prevalence of Hypertension % (95% CI)** | **Risk ratio for** | | |
| --- | --- | --- | --- | --- | --- | --- | --- | --- | --- | --- |
|  |  |  |  | **Mean**  **of age** | **Male %** | **Mortality %** |  | **Death (I^2^%)** | **Severity**  **(I^2^%)** | **ICU admission (I^2^%)** |
| 1. Moula AI, et al: 2020, The Netherlands (1) | 26 | 18/5/20 | 8497(1622) | 58.2±15.6 | 60.3 | 22.8 | 19.1 | 1.73[1.37-2.19] (98) | - | - |
| 1. Silverio A, , et al: 2021, Italy(2) | 45 | 27/4/20 | 18300 | - | - | 12 | - | 1.59; [1.56–1.61] | - | - |
| 1. Mesas AE, et al: 2020, Spain (3) | 60 | 27/7/20 | 51225 (21388) | Ranged between 40 and 73 years | 59.9 | 24.3 | 41.7 | 1.73 [1.09, 2.07] (79) | - | - |
| 1. Soeroto AY, et al: 2020, Indonesia(4) | 16 | 28/7/20 | 6690 | 55.8 | 58 | - | 46.7 | - | - | - |
| 1. Baradaran A, et al: 2020, Iran(5) | 33 | 7/4/20 | 9249 (1675) | 51 | 55 | - | 21 | - | - | - |
| 1. Singh AK, et al: 2020, India(6) | 18 | 23/4/20 | 14558 (1820) | Ranged between 47 to 63 years | 56.2 |  | 22.9 | 1.95 [1.58, 2.41] (92.2) | 1.66 [1.32, 2.09] (30.9) | - |
| 1. Moazzami B, et al: 2020, Iran (7) | 13 | 7/6/20 | 25502 (15268) | 55.25 | 61 | - | 32 | - | - | - |
| 1. de Almeida-Pititto B, et al: 2020, Brazil(8) | 40 | 6/5/20 | 18012 (5160) | - | - | - | 25.4 | 1.95 [1.72–2.73] (35.5) | 1.99 [1.38–2.76] (49.8) | - |
| 1. Biswas M, et al: 2020, Bangladesh (9) | 20 | 21/5/20 | 64676 (6616) | 58.8±16.8 | 55.7 | 5.1 | 11.3 | 1.95[1.58–2.40] (89) | - | - |
| 1. Li B, et al: 2020, China(10) | 6 | ../2/20 | 1527 (198) | 49.7±6.8 | 63 | - | 17.1 | - | 2.03[1.54, 2.68] (41) | - |
| 1. Wong CKH, et al: 2020, China(11) | 76 | 27/3/20 | 11028 (949) | 45.8 (38.6–52.5) | 49.3 |  | 18.1 | - | - | - |
| 1. Krittanawong C, et al: 2020, USA(12) | 13 | 10/4/20 | 49076 (3490) | - | - | - | 7.1 | - | 3.64 [1.86–6.33] (91.7) | - |
| 1. Del Sole F, et al: 2020, Italy(13) | 12 | 28/5/20 | 2794 | weighted median:  50 | 54.6 | - | 19.31 | - | 1.81[1.23- 2.65] | - |
| 1. Barrera FJ, et al: 2020, Mexico(14) | 65 | 6/4/20 | 15794(1416) | ranged from 33 to 75 | 50.2 | - | 17 | 2.39 [1.54-3.73] (66) | 1.48 [0.99-  2.23] (69) | 2.95 [2.18-3.99] (0) |
| 1. Lippi G, et al: 2021, Italy(15) | 13 | 26/3/20 | 2893 | - | - | - | - | 1.96 [1.01–3.40] (0) | 1.98 [1.47–2.62] (24) | - |
| 1. Sreenivasan J, et al: 2020, USA (16) | 10 | 30/3/20 | 1427(497) | 58·5 ± 8.0 | 49.5 | 12.8 | 33.2 | - | 1.54 [1.01-2.05] (74) | - |
| 1. Li J, et al: 2020, China(17) | 12 | 14/4/20 | 2445 | 46 | 56.6 | - | - | - | 1.64 [1.31-2.26] (51.5) | - |
| 1. Hu J and Wang Y: 2021, China(18) | 30 | 26/7/20 | 6685 | - | 60 |  | 34 | - | ‎1.50 [1.04- 2.06] (93.2) | 1.42 [1.11-1.74] (56.8) |
| 1. Yang J, et al: 2020, ‎China(19) | 7 | 25/2/20 | 1576 | median 49.6 | 56.5 | - | 21.1 | - | 1.81  [1.33–2.40] (86.2)‎ | - |
| 1. Zhang J, et al: 2020, ‎China(20) | 18 | 20/3/20 | (4505)-  Severity:2389  Death: 2116 | Severity:50.3  Death: 54.3 | Severity:60.2  Death: 57 | 17.72 | - | 2.40 [1.53–3.48] (56) | 2.27 [1.80–2.86] (8) | - |
| 1. Nandy K, et al: 2020, India(21) | 16 | 28/4/20 | 3994 (1861) | 51.3 | 56.6 | - | 24 | - | 2·95 [2.21 - 3.94] (0) | - |
| 1. Miller L.E, et al: 2020, USA(22) | 16 | 31/3/20 | 1832 | median: 53 | 53 | 9.9 | 24 | - | - | - |
| 1. Xu L et ‎al: 2020, ‎China (23) | 20 | 8/3/20 | 4062 (1083) | - | - | - | - | - | 1.74 [1.11- 2.41] (47.1) | - |
| 1. Lu L, et al: 2020, ‎China (24) | 28 | 11/4/20 | 11818 (3342) | range 20-87 | - | 7.7 | - | 2.08 [0.97- 3.77] (68.6) | - | - |
| 1. Momenzadeh M: 2020, Iran (25) | 9 | ../3/20 | 1596 (268) |  | 56 | - | 16 | - | - | - |
| 1. Khan MMA, et al: 2020, ‎Bangladesh (26) | 41 | 1/5/20 | 27670 (6758) | 60.9 ± 7.9 | 60.3 | 17.3 | 39.5 | 1.73 [1-2.64] | - | - |
| 1. Meng M, et al: 2020, ‎China(27) | 35 | 5/7/20 | 8170 (1817) | 33.3 to 68.6 | 61.5 |  | 22.2 | - | 1.77 [1.29-2.40] (65‎) | - |
| 1. Gold MS, ‎ et al: 2020, Canada(28) | 33 | 20/4/20 | 29096 (566) | - | - | 9.44 | 14.3 | - | - | - |
| 1. Mudatsir M, et al: 2020, Indonesia (29) | 19 | 5/4/20 | 3578 (638) | - | - | - | 21.5 | - | 1.80 [1.03-2.37] (75) | - |
| 1. Espinosa OA, et al: 2020, Brazil (30) | 39 | 15/5/20 | 89238 | Ranged 41 to  70 | 57.4 | 4 | 32 | - | - | - |
| 1. Ssentongo P, et al: 2020, USA(31) | 25 | 9/7/20 | 65484 | 61 | 57 | - | - | 1.82 [1.43- 2.32] (70) | - | - |
| 1. Mahumud RA, et al: 2020, Australia (32) | 23 | 10/4/20 | 202005 | Median:56.4 | 55 | 7 | 22 | - | - | - |
| 1. Pranata R, et al: 2020, Indonesia (33) | 30 | 7/4/20 | (6560) | 50.7 | 55.5 | - | - | 2.21 [1.74- 2.81] (66) | 2.04 [1.69- 2.47] (31) | 2.11 [1.34, 3.33] (18) |
| 1. Parveen R, et al: 2020, India (34) | 7 | 31/3/20 | 2018 (310) | 53.4 | 59 | - | 15.3 | 0.53[0.37- 0.76] (0) | 2.14 [1.22- 3.35] (52.4) | 0.46 [0.24- 0.83] (0) |
| 1. Sales-Peres SHC, et al: 2020, Brazil (35) | 8 | 27/4/20 | 6577 (3388) | 59.7 | 59.8 | - | 51.5 | - | - | - |
| 1. Wu T, et al: 2020, China (36) | 73 | 13/5/20 | 171108 | - | - | 2 | 19 | - | 1.89 [1.57-2.27] (39.2) | - |
| 1. Jain V and Yuan JM: 2020, UK(37) | 7 | 5/3/20 | 1813 | 51.9 | 56.5 | - | In severe group:25.4  In ICU group:40.5 | - | 1.58 [1.01–2.32] | 1.77[1.49-1.99] |
| 1. Chidambaram V, et al: 2020, USA(38) | 109 | 8/5/20 | 38288  (Mortality: 15947)  (Severe disease:7002) | - | - | - | - | 1.90 [1.69–2.15] (28) | 1.86 [1.35–2.56] (64) | - |
| 1. Wang B, et al: 2020, China (39) | 6 | 1/3/20 | 1558 | 48 | 57.1 | - | 17.26 | - | 1.85 [1.24- 2.65] (4) | - |
| 1. Tian W, et al: 2020,USA (40) | 14 | 24/4/20 | 4659 | 59.8 | 57.5 | 25.5 | 43.6 | 1.52 [1.1-2.1] (15) | - | - |
| 1. Wang X, et al: 2020, China (41) | 34 | 6/4/20 | 6263 | - | - | - | - | - | 1.97 [1.40- 2.65] | - |
| 1. Li X, et al: 2020, China (42) | 10 | 14/4/20 | 3118 | Ranged: 49 to 68 | Ranged: 45 to 67 | 16.3 | 24.3 | 2.23[1.75-2.70] (57.4) | - | - |
| 1. Hu Y, et al: 2020, China (43) | 21 | 10/3/20 | 47344 | 51.5 | 40 | 3.2 | 15.6 | - | - | - |
| 1. Zhou Y, et al: 2020, China (44) | 34 | 25/4/20 | 12227 | 52.3 | - | - | 40 | 1.74[1.55-1.82] (55.6) | 1.61 [1.55- 1.82] (48.7) | 1.95 [1.25-2.31] (69.7) |
| 1. Wang Z, et al: 2020, China(45) | 25 | 16/3/20 | 4881 | 43.5 | 45.3 | 14.9 | severe case: 33.4  non-severe cases: 21.6 | - | 1.40[ 1.22–1.60] | - |
| 1. Bae S, et al: 2021, Korea (46) | 51 | 11/6/21 | 48317 | Median: 56 | 58.1 | - | 26.19 | 1.83[1.63- 2.03] | 1.76 [1.57- 1.96] | - |
| 1. Du Y, et al: 2021, China (47) | 24 | 17/11/20 | 99918 | ranged from 40 to 69 years | ranged from 45% to 85%. | - | severe case: 37  nonsurvivors :46 | 1.49 [1.34- 1.62]  (67.3) | 1.70 [1.54- 1.86] (54.1) | - |
| 1. Honardoost M, et al: 2021, Iran (48) | 28 | 4/2/20 | 6270 | Median:63 | 68.8 |  | 20.9 | - | 1.84 [1.54-2.16]  (58.6) | - |
| 1. Li X, et al: 2021, China (49) | 41 | - | 21060 | - | - | - | - | - | 1.78[1.61- 1.95]  (67.9) | - |
| 1. Mishra P, et al: 2021, India (50) | 19 | 5/9/20 | 6872 | - | 56 | - | - | 1.74[1.43- 2.10] | 1.61[1.18- 2] | - |
| 1. Rahman A and Sathi NJ : 2021, Bangladesh(51) | 10 | 18/4/20 | 2272 | - | 60 | - | 21.3 | - | 1.79[1.57-2.04] | - |
| 1. Wu Y, et al: 2021, China (52) | 80 | 26/5/20 | 25385 | - | - | 14 | - | 1.88[1.63- 2.13] | - | - |

1. Moula AI, Micali LR, Matteucci F, Lucà F, Rao CM, Parise O, et al. Quantification of Death Risk in Relation to Sex, Pre-Existing Cardiovascular Diseases and Risk Factors in COVID-19 Patients: Let's Take Stock and See Where We Are. Journal of clinical medicine. 2020;9(9).

2. Silverio A, Di Maio M, Citro R, Esposito L, Iuliano G, Bellino M, et al. Cardiovascular risk factors and mortality in hospitalized patients with COVID-19: systematic review and meta-analysis of 45 studies and 18,300 patients. BMC cardiovascular disorders. 2021;21(1):23.

3. Mesas AE, Cavero-Redondo I, Álvarez-Bueno C, Sarriá Cabrera MA, Maffei de Andrade S, Sequí-Dominguez I, et al. Predictors of in-hospital COVID-19 mortality: A comprehensive systematic review and meta-analysis exploring differences by age, sex and health conditions. PloS one. 2020;15(11):e0241742.

4. Soeroto AY, Soetedjo NN, Purwiga A, Santoso P, Kulsum ID, Suryadinata H, et al. Effect of increased BMI and obesity on the outcome of COVID-19 adult patients: A systematic review and meta-analysis. Diabetes & metabolic syndrome. 2020;14(6):1897-904.

5. Baradaran A, Ebrahimzadeh MH, Baradaran A, Kachooei AR. Prevalence of Comorbidities in COVID-19 Patients: A Systematic Review and Meta-Analysis. The archives of bone and joint surgery. 2020;8(Suppl 1):247-55.

6. Singh AK, Gillies CL, Singh R, Singh A, Chudasama Y, Coles B, et al. Prevalence of co-morbidities and their association with mortality in patients with COVID-19: A systematic review and meta-analysis. Diabetes, obesity & metabolism. 2020;22(10):1915-24.

7. Moazzami B, Chaichian S, Kasaeian A, Djalalinia S, Akhlaghdoust M, Eslami M, et al. Metabolic risk factors and risk of Covid-19: A systematic review and meta-analysis. PloS one. 2020;15(12):e0243600.

8. de Almeida-Pititto B, Dualib PM, Zajdenverg L, Dantas JR, de Souza FD, Rodacki M, et al. Severity and mortality of COVID 19 in patients with diabetes, hypertension and cardiovascular disease: a meta-analysis. Diabetology & metabolic syndrome. 2020;12:75.

9. Biswas M, Rahaman S, Biswas TK, Haque Z, Ibrahim B. Association of Sex, Age, and Comorbidities with Mortality in COVID-19 Patients: A Systematic Review and Meta-Analysis. Intervirology. 2020:1-12.

10. Li B, Yang J, Zhao F, Zhi L, Wang X, Liu L, et al. Prevalence and impact of cardiovascular metabolic diseases on COVID-19 in China. Clinical research in cardiology : official journal of the German Cardiac Society. 2020;109(5):531-8.

11. Wong CKH, Wong JYH, Tang EHM, Au CH, Wai AKC. Clinical presentations, laboratory and radiological findings, and treatments for 11,028 COVID-19 patients: a systematic review and meta-analysis. Scientific reports. 2020;10(1):19765.

12. Krittanawong C, Virk HUH, Narasimhan B, Wang Z, Narasimhan H, Zhang HJ, et al. Coronavirus disease 2019 (COVID-19) and cardiovascular risk: A meta-analysis. Progress in cardiovascular diseases. 2020;63(4):527-8.

13. Del Sole F, Farcomeni A, Loffredo L, Carnevale R, Menichelli D, Vicario T, et al. Features of severe COVID-19: A systematic review and meta-analysis. European journal of clinical investigation. 2020;50(10):e13378.

14. Barrera FJ, Shekhar S, Wurth R, Moreno-Pena PJ, Ponce OJ, Hajdenberg M, et al. Prevalence of Diabetes and Hypertension and Their Associated Risks for Poor Outcomes in Covid-19 Patients. Journal of the Endocrine Society. 2020;4(9):bvaa102.

15. Lippi G, Wong J, Henry BM. Hypertension in patients with coronavirus disease 2019 (COVID-19): a pooled analysis. Polish archives of internal medicine. 2020;130(4):304-9.

16. Sreenivasan J, Khan MS, Anker SD, Kaul R, Khan SU, Metra M, et al. Cardiovascular Risk Factors and Complications in Patients Infected with COVID-19: A Systematic Review. Available at SSRN 3569855. 2020.

17. Li J, He X, Yuan Y, Zhang W, Li X, Zhang Y, et al. Meta-analysis investigating the relationship between clinical features, outcomes, and severity of severe acute respiratory syndrome coronavirus 2 (SARS-CoV-2) pneumonia. American journal of infection control. 2021;49(1):82-9.

18. Hu J, Wang Y. The Clinical Characteristics and Risk Factors of Severe COVID-19. Gerontology. 2021:1-12.

19. Yang J, Zheng Y, Gou X, Pu K, Chen Z, Guo Q, et al. Prevalence of comorbidities and its effects in patients infected with SARS-CoV-2: a systematic review and meta-analysis. International journal of infectious diseases : IJID : official publication of the International Society for Infectious Diseases. 2020;94:91-5.

20. Zhang J, Wu J, Sun X, Xue H, Shao J, Cai W, et al. Association of hypertension with the severity and fatality of SARS-CoV-2 infection: A meta-analysis. Epidemiology and infection. 2020;148:e106.

21. Nandy K, Salunke A, Pathak SK, Pandey A, Doctor C, Puj K, et al. Coronavirus disease (COVID-19): A systematic review and meta-analysis to evaluate the impact of various comorbidities on serious events. Diabetes & metabolic syndrome. 2020;14(5):1017-25.

22. Miller LE, Bhattacharyya R, Miller AL. Diabetes mellitus increases the risk of hospital mortality in patients with Covid-19: Systematic review with meta-analysis. Medicine. 2020;99(40):e22439.

23. Xu L, Mao Y, Chen G. Risk factors for 2019 novel coronavirus disease (COVID-19) patients progressing to critical illness: a systematic review and meta-analysis. Aging. 2020;12(12):12410-21.

24. Lu L, Zhong W, Bian Z, Li Z, Zhang K, Liang B, et al. A comparison of mortality-related risk factors of COVID-19, SARS, and MERS: A systematic review and meta-analysis. The Journal of infection. 2020;81(4):e18-e25.

25. Momenzadeh M. Prevalence of diabetes, hypertension and cardiovascular disease in patients with COVID-19: a systematic review and meta-analysis. EurAsian Journal of BioSciences. 2020;14(1):2195-200.

26. Khan MMA, Khan MN, Mustagir MG, Rana J, Islam MS, Kabir MI. Effects of underlying morbidities on the occurrence of deaths in COVID-19 patients: A systematic review and meta-analysis. Journal of global health. 2020;10(2):020503.

27. Meng M, Zhao Q, Kumar R, Bai C, Deng Y, Wan B. Impact of cardiovascular and metabolic diseases on the severity of COVID-19: a systematic review and meta-analysis. Aging. 2020;12(22):23409-21.

28. Gold MS, Sehayek D, Gabrielli S, Zhang X, McCusker C, Ben-Shoshan M. COVID-19 and comorbidities: a systematic review and meta-analysis. Postgraduate medicine. 2020;132(8):749-55.

29. Mudatsir M, Fajar JK, Wulandari L, Soegiarto G, Ilmawan M, Purnamasari Y, et al. Predictors of COVID-19 severity: a systematic review and meta-analysis. F1000Research. 2020;9:1107.

30. Espinosa OA, Zanetti ADS, Antunes EF, Longhi FG, Matos TA, Battaglini PF. Prevalence of comorbidities in patients and mortality cases affected by SARS-CoV2: a systematic review and meta-analysis. Revista do Instituto de Medicina Tropical de Sao Paulo. 2020;62:e43.

31. Ssentongo P, Ssentongo AE, Heilbrunn ES, Ba DM, Chinchilli VM. Association of cardiovascular disease and 10 other pre-existing comorbidities with COVID-19 mortality: A systematic review and meta-analysis. PloS one. 2020;15(8):e0238215.

32. Mahumud RA, Kamara JK, Renzaho AMN. The epidemiological burden and overall distribution of chronic comorbidities in coronavirus disease-2019 among 202,005 infected patients: evidence from a systematic review and meta-analysis. Infection. 2020;48(6):813-33.

33. Pranata R, Lim MA, Huang I, Raharjo SB, Lukito AA. Hypertension is associated with increased mortality and severity of disease in COVID-19 pneumonia: A systematic review, meta-analysis and meta-regression. Journal of the renin-angiotensin-aldosterone system : JRAAS. 2020;21(2):1470320320926899.

34. Parveen R, Sehar N, Bajpai R, Agarwal NB. Association of diabetes and hypertension with disease severity in covid-19 patients: A systematic literature review and exploratory meta-analysis. Diabetes research and clinical practice. 2020;166:108295.

35. Sales-Peres SHC, de Azevedo-Silva LJ, Bonato RCS, Sales-Peres MC, Pinto A, Santiago Junior JF. Coronavirus (SARS-CoV-2) and the risk of obesity for critically illness and ICU admitted: Meta-analysis of the epidemiological evidence. Obesity research & clinical practice. 2020;14(5):389-97.

36. Wu T, Zuo Z, Kang S, Jiang L, Luo X, Xia Z, et al. Multi-organ Dysfunction in Patients with COVID-19: A Systematic Review and Meta-analysis. Aging and disease. 2020;11(4):874-94.

37. Jain V, Yuan JM. Predictive symptoms and comorbidities for severe COVID-19 and intensive care unit admission: a systematic review and meta-analysis. International journal of public health. 2020;65(5):533-46.

38. Chidambaram V, Tun NL, Haque WZ, Majella MG, Sivakumar RK, Kumar A, et al. Factors associated with disease severity and mortality among patients with COVID-19: A systematic review and meta-analysis. PloS one. 2020;15(11):e0241541.

39. Wang B, Li R, Lu Z, Huang Y. Does comorbidity increase the risk of patients with COVID-19: evidence from meta-analysis. Aging. 2020;12(7):6049-57.

40. Tian W, Jiang W, Yao J, Nicholson CJ, Li RH, Sigurslid HH, et al. Predictors of mortality in hospitalized COVID-19 patients: A systematic review and meta-analysis. Journal of medical virology. 2020;92(10):1875-83.

41. Wang X, Fang X, Cai Z, Wu X, Gao X, Min J, et al. Comorbid Chronic Diseases and Acute Organ Injuries Are Strongly Correlated with Disease Severity and Mortality among COVID-19 Patients: A Systemic Review and Meta-Analysis. Research (Washington, DC). 2020;2020:2402961.

42. Li X, Guan B, Su T, Liu W, Chen M, Bin Waleed K, et al. Impact of cardiovascular disease and cardiac injury on in-hospital mortality in patients with COVID-19: a systematic review and meta-analysis. Heart (British Cardiac Society). 2020;106(15):1142-7.

43. Hu Y, Sun J, Dai Z, Deng H, Li X, Huang Q, et al. Prevalence and severity of corona virus disease 2019 (COVID-19): A systematic review and meta-analysis. Journal of clinical virology : the official publication of the Pan American Society for Clinical Virology. 2020;127:104371.

44. Zhou Y, Yang Q, Chi J, Dong B, Lv W, Shen L, et al. Comorbidities and the risk of severe or fatal outcomes associated with coronavirus disease 2019: A systematic review and meta-analysis. International journal of infectious diseases : IJID : official publication of the International Society for Infectious Diseases. 2020;99:47-56.

45. Wang Z, Deng H, Ou C, Liang J, Wang Y, Jiang M, et al. Clinical symptoms, comorbidities and complications in severe and non-severe patients with COVID-19: A systematic review and meta-analysis without cases duplication. Medicine. 2020;99(48):e23327.

46. Bae S, Kim SR, Kim MN, Shim WJ, Park SM. Impact of cardiovascular disease and risk factors on fatal outcomes in patients with COVID-19 according to age: a systematic review and meta-analysis. Heart (British Cardiac Society). 2021;107(5):373-80.

47. Du Y, Zhou N, Zha W, Lv Y. Hypertension is a clinically important risk factor for critical illness and mortality in COVID-19: A meta-analysis. Nutrition, metabolism, and cardiovascular diseases : NMCD. 2021;31(3):745-55.

48. Honardoost M, Janani L, Aghili R, Emami Z, Khamseh ME. The Association between Presence of Comorbidities and COVID-19 Severity: A Systematic Review and Meta-Analysis. Cerebrovascular diseases (Basel, Switzerland). 2021;50(2):132-40.

49. Li X, Zhong X, Wang Y, Zeng X, Luo T, Liu Q. Clinical determinants of the severity of COVID-19: A systematic review and meta-analysis. PloS one. 2021;16(5):e0250602.

50. Mishra P, Parveen R, Bajpai R, Samim M, Agarwal NB. Impact of cardiovascular diseases on severity of COVID-19 patients: A systematic review. Annals of the Academy of Medicine, Singapore. 2021;50(1):52-60.

51. Rahman A, Sathi NJ. Risk factors of the severity of COVID-19: A meta-analysis. International journal of clinical practice. 2021;75(7):e13916.

52. Wu Y, Li H, Zhang Z, Liang W, Zhang T, Tong Z, et al. Risk factors for mortality of coronavirus disease 2019 (COVID-19) patients during the early outbreak of COVID-19: a systematic review and meta-analysis. Annals of palliative medicine. 2021;10(5):5069-83.
