# Supplementary material for: Prevalence of hypertension and associated risks in hospitalized patients with COVID-19: a meta-analysis of meta-analyses with 1468 studies and 1,281,510 patients
Source: Syst Rev. 2022 Nov 17;11:242. doi: 10.1186/s13643-022-02111-2 (PMC9672558; doi:10.1186/s13643-022-02111-2)
Supplement: Supplementary file 4 — Additional file 4. AMSTAR (Assessment of Multiple Systematic Reviews) checklist. [file 13643_2022_2111_MOESM4_ESM.docx]

| Table3-AMSTAR (Assessment of Multiple Systematic Reviews) checklist | | | | | | | | | | | | |
| --- | --- | --- | --- | --- | --- | --- | --- | --- | --- | --- | --- | --- |
| Reference | 1 | 2 | 3 | 4 | 5 | 6 | 7 | 8 | 9 | 10 | 11 | Score from11 |
| Moula AI, et al:2020, The Netherlands | No | Yes | Yes | Yes | No | Yes | Yes | Yes | Yes | Yes | Yes | 9 |
| Emami A, et al:2020, Iran. | No | No | No | No | No | Yes | No | No | Yes | No | Yes | 3 |
| Silverio A, , et al:2021, Italy | Yes | Yes | Yes | Yes | Yes | No | Yes | Yes | No | Yes | Yes | 9 |
| Mesas AE, , et al:2020, Spain | Yes | Yes | Yes | Yes | Yes | Yes | Yes | Yes | Yes | Yes | CA | 10 |
| Soeroto AY, et al:2020, Indonesia | No | Yes | Yes | Yes | Yes | Yes | No | No | Yes | Yes | Yes | 8 |
| Baradaran A, et al:2020, Iran | No | Yes | Yes | No | Yes | Yes | No | No | Yes | Yes | No | 6 |
| Singh AK, et al:2020, India | Yes | Yes | Yes | Yes | No | Yes | Yes | Yes | Yes | Yes | Yes | 10 |
| Moazzami B, et al:2020, Iran | No | Yes | Yes | Yes | No | Yes | Yes | No | Yes | Yes | CA | 7 |
| de Almeida-Pititto B, et al:2020 | Yes | Yes | Yes | Yes | Yes | Yes | Yes | Yes | Yes | Yes | Yes | 11 |
| Biswas M, et al:2020 | No | Yes | No | Yes | No | Yes | Yes | Yes | Yes | Yes | Yes | 8 |
| Li B, et al:2020 | No | Yes | Yes | Yes | No | Yes | Yes | CA | Yes | Yes | No | 8 |
| Wong CKH, et al:2020 | Yes | Yes | Yes | Yes | Yes | Yes | No | No | Yes | Yes | Yes | 9 |
| Del Sole F, et al:2020, Italy | No | Yes | Yes | CA | Yes | Yes | Yes | No | Yes | No | Yes | 7 |
| Barrera FJ, et al:2020, Mexico | Yes | Yes | Yes | Yes | Yes | Yes | Yes | CA | Yes | Yes | No | 9 |
| Lippi G,‎‏ ‏et al:2021, ‎Italy | No | Yes | Yes | CA | No | Yes | No | No | Yes | No | Yes | 5 |
| ‎Sreenivasan J, ‎et al:2020, ‎USA | Yes | Yes | Yes | Yes | Yes | Yes | Yes | Yes | Yes | Yes | CA | 10 |
| Li J, et al:2020, China | No | Yes | Yes | Yes | Yes | Yes | Yes | Yes | Yes | Yes | No | 9 |
| Hu J and Wang Y, 2020, China | No | Yes | Yes | Yes | Yes | Yes | No | No | Yes | Yes | Yes | 8 |
| ‎Yang J, et ‎al:2020, ‎China | No | Yes | Yes | No | Yes | Yes | No | No | Yes | No | Yes | 6 |
| Zhang J, et ‎al:2020, ‎China | No | Yes | Yes | Yes | Yes | Yes | Yes | CA | Yes | Yes | Yes | 9 |
| Nandy K, et al:2020, ‎India | No | Yes | Yes | Yes | Yes | Yes | Yes | CA | Yes | No | Yes | 8 |
| Miller L.E, et al:2020, ‎USA | Yes | Yes | Yes | Yes | Yes | Yes | No | No | Yes | Yes | Yes | 9 |
| Xu L et ‎al:2020, ‎China | No | CA | CA | CA | Yes | Yes | Yes | No | Yes | Yes | Yes | 6 |
| Lu L, et ‎al:2020, ‎China | Yes | Yes | Yes | No | Yes | Yes | Yes | CA | Yes | Yes | Yes | 9 |
| Momenzadeh M, ‎‎2020, Iran‎ | No | Yes | Yes | No | Yes | Yes | Yes | CA | Yes | No | CA | 6 |
| KhanMMA, et ‎al:2020, ‎Bangladesh | No | Yes | Yes | Yes | Yes | Yes | Yes | Yes | Yes | Yes | Yes | 10 |
| Meng M, et ‎al:2020, ‎China | No | CA | CA | CA | Yes | Yes | Yes | CA | Yes | Yes | Yes | 6 |
| Gold MS, ‎ et al:2020, ‎Canada | No | CA | Yes | Yes | Yes | Yes | Yes | CA | Yes | No | Yes | 7 |
| Mudatsir M, et al:2020, Indonesia | No | CA | Yes | No | Yes | Yes | Yes | Yes | Yes | Yes | Yes | 8 |
| Espinosa OA, et al:2020, Brazil | Yes | Yes | Yes | Yes | Yes | Yes | Yes | CA | Yes | Yes | CA | 9 |
| Ssentongo P, et al:2020, USA | Yes | Yes | Yes | Yes | Yes | Yes | Yes | Yes | Yes | Yes | Yes | 11 |
| Mahumud RA, et al:2020, Australia | No | Yes | Yes | Yes | Yes | Yes | Yes | Yes | Yes | Yes | CA | 9 |
| Pranata R, et al:2020, Indonesia | No | Yes | Yes | Yes | Yes | Yes | No | Yes | Yes | Yes | Yes | 9 |
| Sales-Peres SHC, et al:2020, Brazil | No | Yes | Yes | Yes | No | Yes | Yes | Yes | Yes | Yes | Yes | 9 |
| Wu T, et al:2020, China | Yes | Yes | Yes | Yes | Yes | Yes | Yes | Yes | Yes | Yes | Yes | 11 |
| Jain V and Yuan JM. 2020, UK | Yes | Yes | Yes | Yes | Yes | Yes | Yes | CA | Yes | No | Yes | 9 |
| Chidambaram V, et al:2020, USA | Yes | Yes | Yes | Yes | Yes | Yes | Yes | Yes | Yes | Yes | Yes | 11 |
| Wang B, et al:2020, China | No | Yes | Yes | Yes | Yes | Yes | Yes | Yes | Yes | Yes | Yes | 10 |
| Tian W, et al:2020,USA | No | Yes | Yes | Yes | Yes | Yes | Yes | Yes | Yes | No | Yes | 9 |
| Wang X, et al:2020, China | No | Yes | Yes | No | Yes | Yes | No | Yes | Yes | Yes | Yes | 8 |
| Li X, et al:2020, China | No | Yes | Yes | Yes | Yes | Yes | Yes | Yes | Yes | Yes | Yes | 10 |
| Hu Y, et al:2020, China | No | Yes | Yes | No | Yes | Yes | Yes | Yes | Yes | Yes | Yes | 9 |
| Zhou Y, et al:2020, China | Yes | Yes | Yes | Yes | Yes | Yes | Yes | Yes | Yes | Yes | Yes | 11 |
| Wang Z, et al:2020, China | Yes | Yes | Yes | Yes | Yes | Yes | Yes | Yes | Yes | Yes | Yes | 11 |
| Bae S, et al:2021, Korea | Yes | Yes | Yes | Yes | Yes | Yes | Yes | Yes | Yes | Yes | Yes | 11 |
| Du Y, et al:2021 | Yes | Yes | Yes | Yes | Yes | Yes | Yes | Yes | Yes | Yes | Yes | 11 |
| Honardoost M, et al:2021, Iran | No | CA | Yes | No | Yes | Yes | Yes | No | Yes | Yes | Yes | 8 |
| Li X, et al:2021, China | No | CA | Yes | Yes | Yes | Yes | Yes | CA | No | Yes | Yes | 8 |
| Mishra P, et al:2021, India | Yes | Yes | Yes | Yes | Yes | Yes | Yes | CA | Yes | Yes | CA | 9 |
| Rahman A and Sathi NJ :2021,Bangladesh | No | Yes | Yes | Yes | Yes | Yes | CA | CA | Yes | Yes | Yes | 8 |
| Wu Y, et al:2021, China | Yes | Yes | Yes | No | Yes | Yes | Yes | Yes | Yes | Yes | Yes | 10 |
| 1- Was an “a priori” design provided?, 2- was there duplicate study selection and data extraction?, 3- was a comprehensive literature search performed?, 4- was the status of publication (ie, grey literature) used as an inclusion criterion?, 5- was a list of studies (included and excluded) provided?, 6- were the characteristics of the included studies provided?, 7- Was the scientific quality of the included studies assessed and documented?, 8-Was the scientific quality of the included studies used appropriately in formulating conclusions?, 9- Were the methods used to combine the findings of studies appropriate?, 10- was the likelihood of publication bias assessed?, 11- Was the conflict of interest included?  Y=Yes N=No CA= Can't answer NA= Not applicable | | | | | | | | | | | | |
